# Ginkgetin induces autophagic cell death through p62/SQSTM1-mediated autolysosome formation and redox setting in non-small cell lung cancer

**DOI:** 10.18632/oncotarget.21862

**Published:** 2017-10-16

**Authors:** Jian-Shu Lou, Wen-Chuan Bi, Gallant K.L Chan, Yan Jin, Chau-Wing Wong, Zhong-Yu Zhou, Huai-You Wang, Ping Yao, Tina T.X. Dong, Karl W.K. Tsim

**Affiliations:** ^1^ Shenzhen Research Institute, The Hong Kong University of Science and Technology, Shenzhen, China; ^2^ Division of Life Science, Center for Chinese Medicine, The Hong Kong University of Science and Technology, Clear Water Bay, Hong Kong, China

**Keywords:** autophagy, natural compounds, non-small cell lung cancer, p62, mTORC1

## Abstract

Promoting cell death by autophagy could be a novel treatment for cancer. The major player in autophagy, p62, serves as a good therapeutic target. Ginkgetin, a biflavonoid from *Ginkgo biloba* leaves, exhibited promising anticancer activity in non-small cell lung cancer cell lines, with an IC50 lower than that of cisplatin. This anticancer effect of ginkgetin was illustrated in a xenograft nude mouse model. Ginkgetin induced autophagic cell death in A549 cells, and this effect was markedly reversed by chemical and genetic approaches. Ginkgetin showed potential binding affinity to p62. Upregulation of p62 through chemical and genetic means decreased cell death, lysosome acidification, and autophagosome formation, which consequently disrupted autolysosome formation. In addition, the decreased autophagy induced by p62 overexpression increased Nrf2/ARE activity and the oxygen consumption rate and decreased on formation of reactive oxygen species. These phenomena were exhibited in a reciprocal manner when p62 was knocked down. Thus, p62 may be a potential target in ginkgetin-induced autophagic cell death, and ginkgetin could be developed as a novel anticancer drug.

## INTRODUCTION

The International Agency for Research on Cancer estimated that 10 million lung cancer-related deaths would occur per year by 2030. Based on histopathology, non-small cell lung cancer (NSCLC) is a major type of lung cancer; it accounts for almost 80% of lung cancers and exhibits intrinsic resistance to anticancer drugs, including platinum-based antineoplastic drugs [1, 2] and epidermal growth factor receptor tyrosine kinase inhibitors (EGFR-TKIs) [3, 4]. Such challenges have shifted the focus of research to finding novel drugs that may concomitantly target multiple mechanisms and would therefore enhance tumor regression.

The majority of chemotherapeutic drugs exhibit cytotoxic action via apoptosis; however, cancer cells may evade cell death through either intrinsic resistance or late-acquired resistance via genetic and epigenetic modifications. Most chemotherapeutic drugs share common apoptotic pathways; however, a simple mutation that disables apoptosis can trigger multidrug resistance [5]. This multidrug resistance issue in cancer therapy is therefore necessitating a search for alternate pathways of cell death that can be targeted independently or concomitantly with apoptotic signaling pathways to elicit effective and sustained inhibition of cancer growth [6]. Under this scenario, cancer cell autophagy has received much attention in recent years as a potent mechanism to overcome multidrug resistance.

Autophagy is a self-digestive process wherein misfolded and aggregated proteins along with damaged organelles are sequestered by double-membrane vesicles called autophagosomes. An autophagosome is delivered to a lysosome to form an autolysosome for subsequent degradation and recycling. However, the effect of autophagy on cell fate in the context of cancer therapy remains controversial [7–9]. The major objection to using autophagy as a cancer therapy is the possible supply of nutrients it could produce for cancer cells through the recycling process [10]. In contrast, malfunctions of autophagy eventually lead to excessive self-degradation of cellular components that are essential for survival, eventually driving cell death [11]. The use of autophagy-induced cell death is proposed as a novel treatment for cancer, including the development of novel drugs that aggravate apoptosis through induction of autophagy [12].

p62 is a major player in autophagy and serves as a signaling hub for several signal transduction pathways. The development of tumors in lung cancer is highly dependent on p62; for example, the development of cancer can be delayed by knocking out p62 in several mouse models [13, 14]. A p62-encoding DNA vaccine has been reported to exhibit encouraging anticancer effect in several animal models, including mouse, rat and dog, without significant side effects. Thus, p62 serves as a good therapeutic target, especially in special oncogenes are undruggable or target therapy yield resistance [15, 16].

Phytochemicals are crucial in developing anticancer drugs. Approximately 62% of anticancer drugs approved by the FDA from 1981 to 2002 were isolated from natural resources, and they are either completely or semi-synthesized based on their natural structure [17]. For instance, Taxol derived from *Taxus* plants (yew) has been successfully used in clinics as a standard therapy for prostate and breast cancer patients. Natural compounds usually have autophagic functions, and p62-targeted therapy merits further clinical development. Thus, the search for anticancer drugs from phytochemicals that target autophagic proteins has become a popular trend.

Ginkgetin, a bioflavonoid originating from *Ginkgo biloba* leaves, exhibits anti-inflammatory, anti-influenza, and neuroprotective activities [18–20]. The anti-cancer effect of ginkgetin has been recently reported (e.g., an anti-tumor effect on a prostate cancer cell line) [21]. However, the mechanism by which ginkgetin performs this antitumor function remains unclear. In addition, the role of ginkgetin in inducing cell death via autophagy has not been reported. The present study revealed that ginkgetin mediated autophagic cell death in NSCLC A549 cells through induction of autolysosome formation and redox setting, and revealed the key role of p62 in this process.

## RESULTS

### Ginkgetin induces cell death and disrupts mitochondrial function

**Figure 1:**
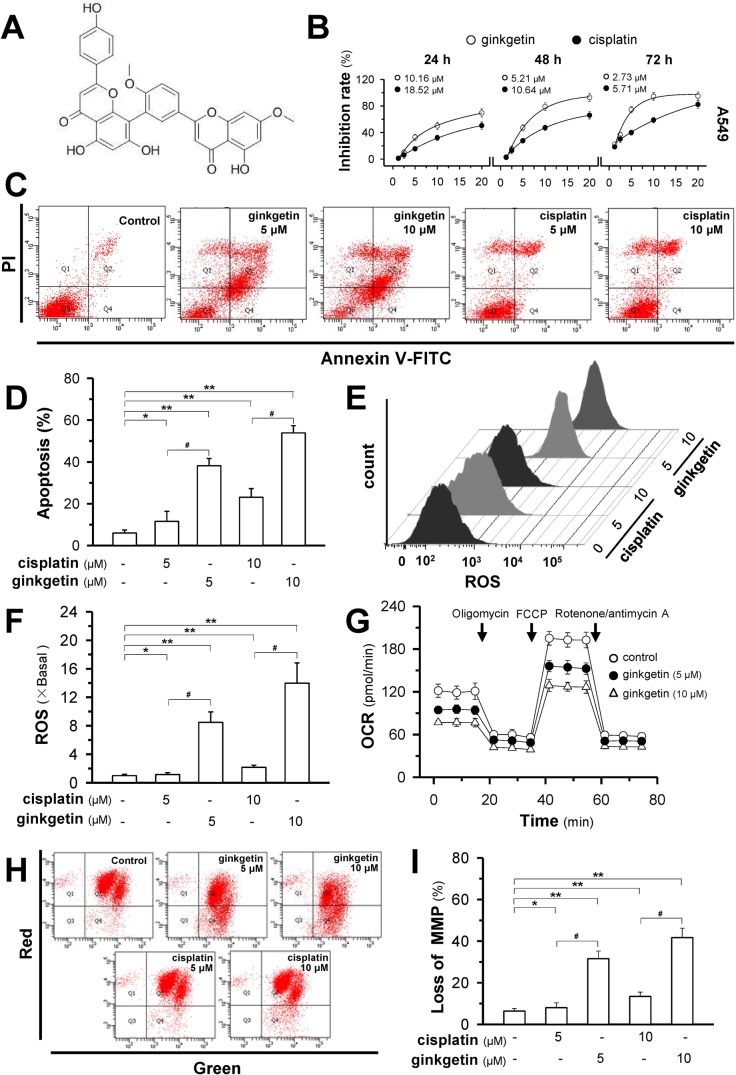
Ginkgetin induces cell death and mitochondria disruption in non-small cell lung cancer **(A)** Chemical structure of ginkgetin. **(B)** Cytotoxicity of ginkgetin was observed in A549 cell line. Cells were seeded in 96-well plates (3 × 10^3^ cells/well) and subsequently were treated with ginkgetin or cisplatin for 24h, 48h and 72 hours. MTT assay was employed to reveal the cell survival. The IC_50_ in different scenarios are shown. Values are in percentage of cell growth inhibition. **(C)** Cultured A549 cells were treated with ginkgetin and cisplatin at different concentrations for 48 hours. The dual parametric dot plots combining annexin V-FITC and PI fluorescence showed viable cell population in bottom left quadrant (Q3), the early apoptotic cells in bottom right quadrant (Q4), and the late apoptotic cells in top right quadrant (Q2). **(D)** Apoptotic rates were calibrated from (C). Values are in percentage of apoptotic cell number. **(E)** Cells were treated as in (C). The amount of ROS was detected by a flow cytometry. **(F)** Mean fluorescence density of ROS level was calibrated from (E). **(G)** Oxygen consumption rate (OCR) in ginkgetin-treated A549 cells after 6 hours. OCR measurements were performed with Mito Stress Test Kit with Seahorse instrument. **(H)** Cells were treated as in (C). The MMP was detected by a flow cytometry. **(I)** The percentage of MMP loss was calibrated from (H). Values are in percentage of MMP loss as compared to control (no drug treatment). Results are expressed as mean ± SEM from three separate experiments, *n* = 6. ^*^p < 0.05, ^**^p < 0.01, versus control. ^#^ *p* < 0.05, versus cisplatin-treated.

Ginkgetin is a biflavonoid (Figure 1A). The cytotoxicity effect of ginkgetin was illustrated in three cancer cell lines, A549 (EGFR, PIK3CA, p52 functional), PC9 (EGFR mutant) and NCIH-460 (EGFR, p52 functional, PIK3CA mutant). Ginkgetin more potently inhibited cell proliferation in all cell lines compared to the standard anti-cancer drug cisplatin, with lower IC50 values (Figure 1B and Supplementary Figure 1). To further investigate the molecular and cellular effects of ginkgetin, the most sensitive cell line, A549, was chosen for further analysis. As expected, ginkgetin-induced apoptosis was approximately three-fold higher than that induced by cisplatin at the same dose (Figure 1C and 1D). Multiple chemotherapeutic and non-chemotherapeutic modalities will cause dysfunctions in mitochondria. Here, ginkgetin-induced cell death showed a robust increase in reactive oxygen species (ROS) which was much higher than that following cisplatin treatment (Figure 1E and 1F). Along with the increase in ROS, an approximately 1.5 to 1.8-fold decrease in the oxygen consumption rate (OCR) was observed in ginkgetin-treated A549 cells (Figure 1G). In parallel, the decline in mitochondria membrane potential (MMP), as determined by flow cytometry, was notably increased after treatment with ginkgetin in A549 cells (Figure 1H and 1I).

### Ginkgetin induces autophagy

**Figure 2:**
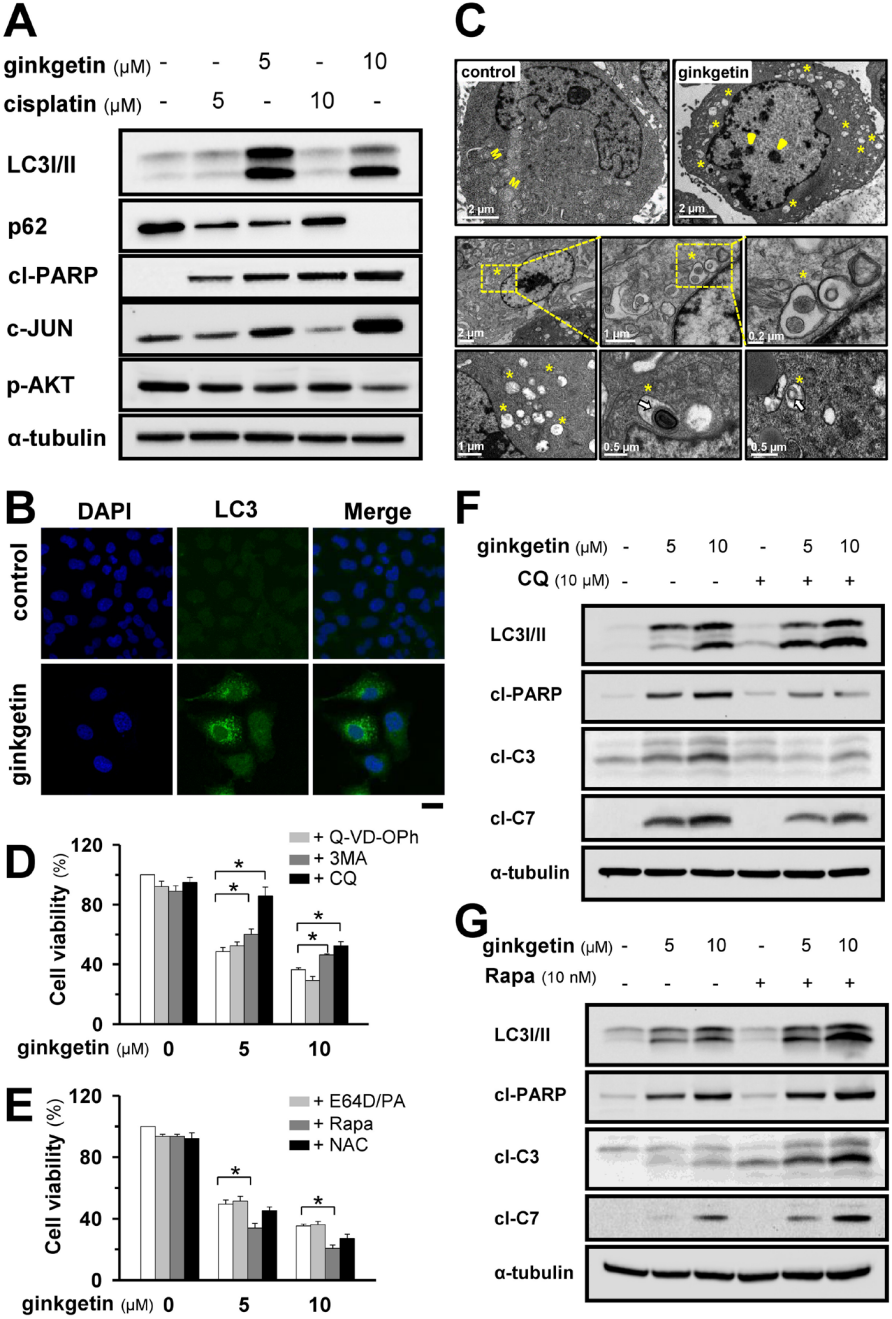
Ginkgetin induces autophagy responsible for cell death in A549 **(A)** A549 cells were treated with ginkgetin and cisplatin at different doses for 48 hours. The protein expressions of LC3 I/II (∼14 and ∼16 kDa), p62 (∼62 kDa), cleaved-PARP (cl-PARP; ∼89 kDa), c-JUN (∼43 kDa), p-AKT (∼60 kDa) were determined by western blotting. Expression of α-tubulin (∼55 kDa) served as a control. **(B)** A549 cells were treated with ginkgetin (10 μM) for 48 hours, cells were fixed and stained with anti-LC3 (green), and nuclei staining by DAPI (blue), observed by a confocal microscope. Bar = 20 μm. Representative photos were shown. **(C)** Autophagy identification by transmission electron microscopy. A549 cells were treated with ginkgetin (10 μM) for 24 hours. Yellow asterisk indicates the autophagical vacuoles, and yellow arrowhead shows the chromosome condensation in nucleus. M represents mitochondria in cytoplasm. White arrow indicates the organelle remnants. Different views at different magnification were shown. **(D)** A549 cells were seeded in 96-well plates (3 × 10^3^ cells/well), allowed to adhere overnight and subsequently were treated with ginkgetin for 48 hours, with or without apoptosis inhibitor Q-VD-OPh (20 μM) and autophagy inhibitors 3-methyladenine (3-MA; 1 mM) and chloroquine (CQ; 10 μM). Cell toxicity was observed. **(E)** Ginkgetin was applied as in (D) with or without E64D/PA (10 μM), rapamycin (Rapa; 10 nM) and N-acetyl-L-cystein (NAC; 20 μM). Cell toxicity was observed. **(F)** Ginkgetin, with or without chloroquine (10 μM), and **(G)** Ginkgetin, with or without Rapamycin (10 nM). Amounts of LC3 I/II, cleaved-PARP (cl-PARP; ∼89 kDa), cleaved-caspase 3 (cl-C3; ∼17 and ∼19 kDa) and 7 (cl-C7; ∼20 kDa) were detected by western blot. Expression of α-tubulin (∼55 kDa) served as a control. Values are in percentage of cell growth. Each point represents mean ± SEM, *n* = 3. ^*^p < 0.05.

Ginkgetin markedly increased the levels of the autophagy marker LC3 I/II and decreased the level of p62 in a dose-dependent (Figure 2A) and time-dependent (Supplementary Figure 2) manners. The elevation of LC3 I/II was further confirmed by immunostaining (Figure 2B). The c-JUN N-terminal kinase is known to control autophagy, and the activation of c-JUN not only leads to apoptotic cell death but also induces autophagic cell death [22]. Here, the increases in LC3 I/II and c-JUN induced by ginkgetin occurred concomitant with an increase in the apoptotic marker cleaved-PARP (Figure 2A). By contrast, the cisplatin-induced increase in cleaved-PARP was not accompanied by increases in LC3 I/II and c-JUN (Figure 2A). As expected, the level of phosphorylated AKT was decreased in drug-treated A549 cells. Ginkgetin-induced autophagy was further confirmed by transmission electronic microscopy. Autophagic vesicles were clearly detected in the cytoplasm of ginkgetin-treated cells. The majority of autophagic vesicles were revealed to have double-membrane structures with an average diameter of 0.5–1.5 μm (Figure 2C). Thus, the administration of ginkgetin in cultured A549 cells could trigger both autophagy and apoptosis.

To confirm the major cell death pathway functioning in ginkgetin-treated cells, inhibitors of autophagy and apoptosis were applied. The apoptosis inhibitor Q-VD-OPh did not reverse the ginkgetin-induced cell death (Figure 2D). However, the application of 3-methyladenine, a blocker of autophagosome formation, rescued ginkgetin-induced cell death. Similarly, chloroquine, a 4-aminoquinoline compound that inhibits lysosomal acidification and autophagosome degradation, reversed ginkgetin-induced cell toxicity (Figure 2D). By contrast, the application of E64D plus pepstatin A (E64D/PA, 1:1), which inhibits lysosomal enzymes, did not rescue ginkgetin-induced cell death. The autophagy inducer rapamycin notably enhanced the toxicity of ginkgetin (Figure 2E). Considering that robust ROS formation was induced by ginkgetin, we administered a ROS scavenger, N-acetyl-L-cysteine, to ginkgetin-treated cells. Unexpectedly, the ginkgetin-induced toxicity in A549 cells was not reversed (Figure 2E). This result indicated that ginkgetin-induced cell death could be mediated by autophagy and that ROS formation might be a consequent event.

Autophagy blocking by chloroquine was confirmed by the elevation of LC3 I/II. Consistent with the changes in ginkgetin-induced cytotoxicity after autophagy was blocked, the apoptotic markers cleaved-PARP, cleaved-caspase 3, and cleaved-caspase 7 were significantly decreased with the co-treatment of chloroquine and ginkgetin in cultured A549 cells (Figure 2F). Conversely, the application of rapamycin in ginkgetin-treated A549 cells further increased these apoptotic markers (Figure 2G). These data supported the notion that ginkgetin-induced cell toxicity might largely depend on lysosome acidification and autophagosome formation.

### Ginkgetin suppresses the formation of the p62-mTORC1 complex

**Figure 3:**
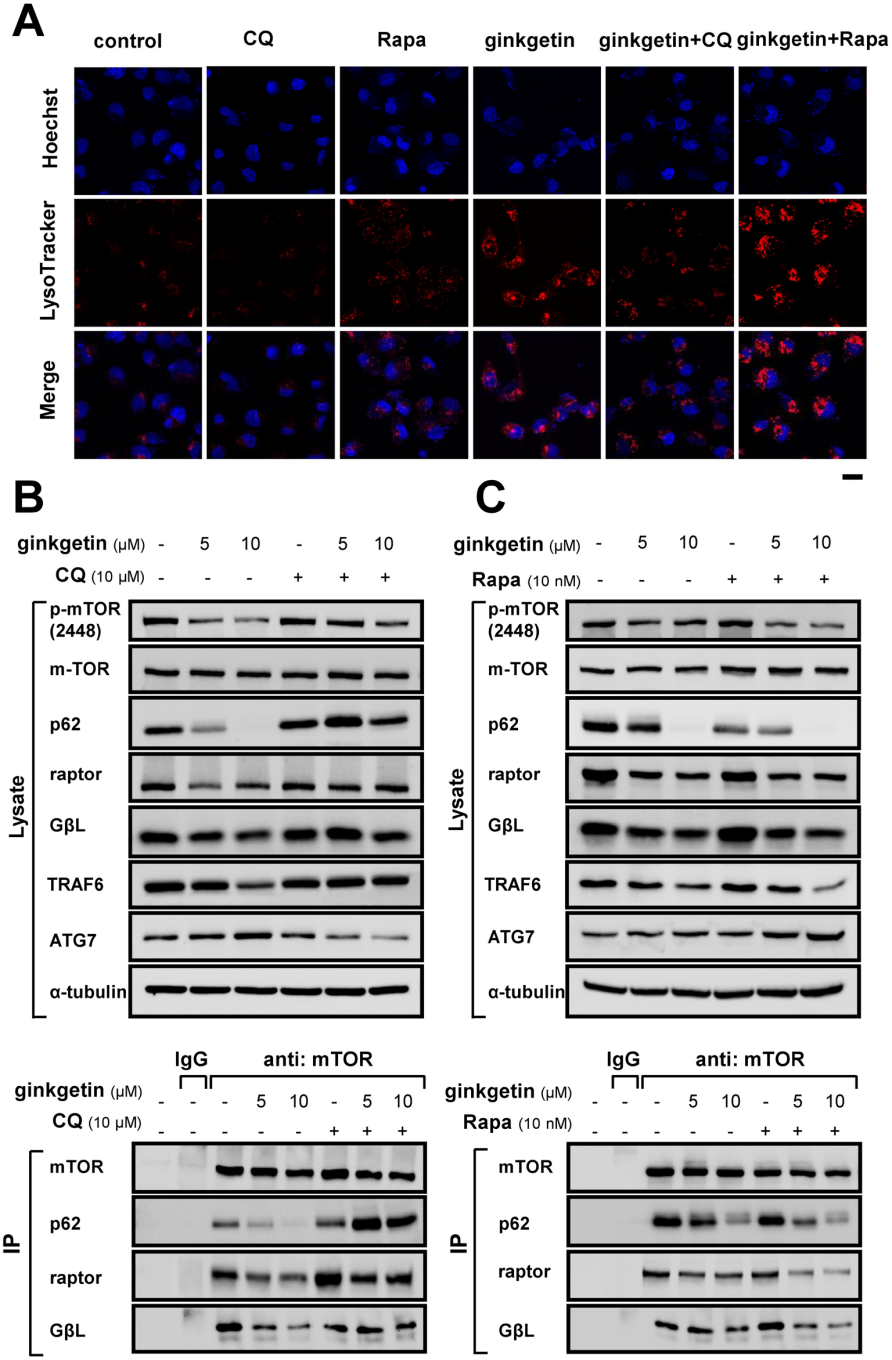
Ginkgetin induces lysosome acidification and disrupts the p62-mTOC1 complex **(A)** A549 cells were seeded in cover glasses and treated with ginkgetin (10 μM) in the presence or absence of chloroquine (CQ; 10 μM), or rapamycin (Rapa; 10 nM), for 12 hours. Cells were stained with LysoTracker® Red DND-99 (100 nM, 1 hour) and Hoechst 33258 (1 μg/ml, 20 min). Cover glasses with cells were fixed and observed under a Zeiss Laser Scanning Confocal Microscope (LSM7 DUO). Bar = 20 μm. A549 cells were treated with ginkgetin with or without chloroquine **(B)**, or rapamycin **(C)**, for 48 hours, followed by immunoprecipitation (IP) with the antibody of mTOR (1 μg) and protein G agarose, IgG (1 μg) was added to lysis of untreated group together with protein G agarose as control, and immunoblotting with specific antibodies (lower panel). Total cell lysates were probed with the antibodies (upper panel). Amounts of p-mTOR (∼289 kDa), mTOR (∼289 kDa), p62 (∼62 kDa), raptor (∼150 kDa), GβL (∼37 kDa), TRAF6 (∼58 kDa), and ATG7 (∼78 kDa) were detected. Expression of α-tubulin (∼55 kDa) served as a control. Representative photo and gel were shown, *n* = 3.

Lysosomes are acidic organelles, and their proper acidification is critical for their proteolyticactivity [23, 24]. The reversing effect of chloroquine on ginkgetin-induced toxicity led us to investigate whether lysosome acidification was altered in live cells. Lysosome acidification was visualized in live cells by staining acidic organelles with LysoTracker Red dye. In naïve cultured A549 cells, the LysoTracker Red staining was weak and diffuse. After ginkgetin application, intense and punctate LysoTracker Red staining was observed. In parallel, chloroquine could reverse ginkgetin-induced staining, while rapamycin could promote such staining (Figure 3A).

Activation of lysosomal function is associated with the formation of the p62-mTORC1 complex, which is critical for autophagosome-lysosome fusion and occurs via TRAF6-mediated K63-linked ubiquitination of mTORC1 and p62 [25–27]. Here, in total cell lysate, the phosphorylation of mTOR at ser-2448, as well as TRAF6 was decreased by treatment with ginkgetin (Figure 3B and 3C), and this decrease was rescued by chloroquine, which may indicate that decreases in mTORC1 activity and p62-mTORC1 formation accompany autophagy induction [28]. Next, various components of the p62-mTORC1 complex were determined by immunoprecipitation (IP) using anti-mTOR antibody.

The amount of p62 in the total lysate was markedly decreased in the presence of ginkgetin, while its association with mTORC1, as p62 immunoprecipitated together with mTOR, was further decreased (Figure 3B). However, the co-application of chloroquine blocked the reduction in p62 in the cell lysate and in the IP product. The mTOR-associated proteins raptor and GβL slightly decreased in the total cell lysate after ginkgetin exposure but significantly decreased in IP products, and these effects were reversed by the administration of chloroquine. (Figure 3B). By contrast, the autophagy inducer rapamycin further decreased p62 in the total cell lysate and IP products, and decreased the interactions of raptor and GβL with mTOR (Figure 3C). In addition, ATG7 modulates lysosome acidification via autophagasome-lysosome fusion [29]. An increase in ATG7 was observed in ginkgetin-treated cells, and this increase was reversed by chloroquine but promoted by rapamycin (Figure 3B and 3C). These results indicated that ginkgetin could disrupt the p62-mTORC1 complex and increase ATG7, which might subsequently increase autophagasome-lysosome fusion and lysosome activity.

### Ginkgetin-induced autophagosome formation is mediated by p62

**Figure 4:**
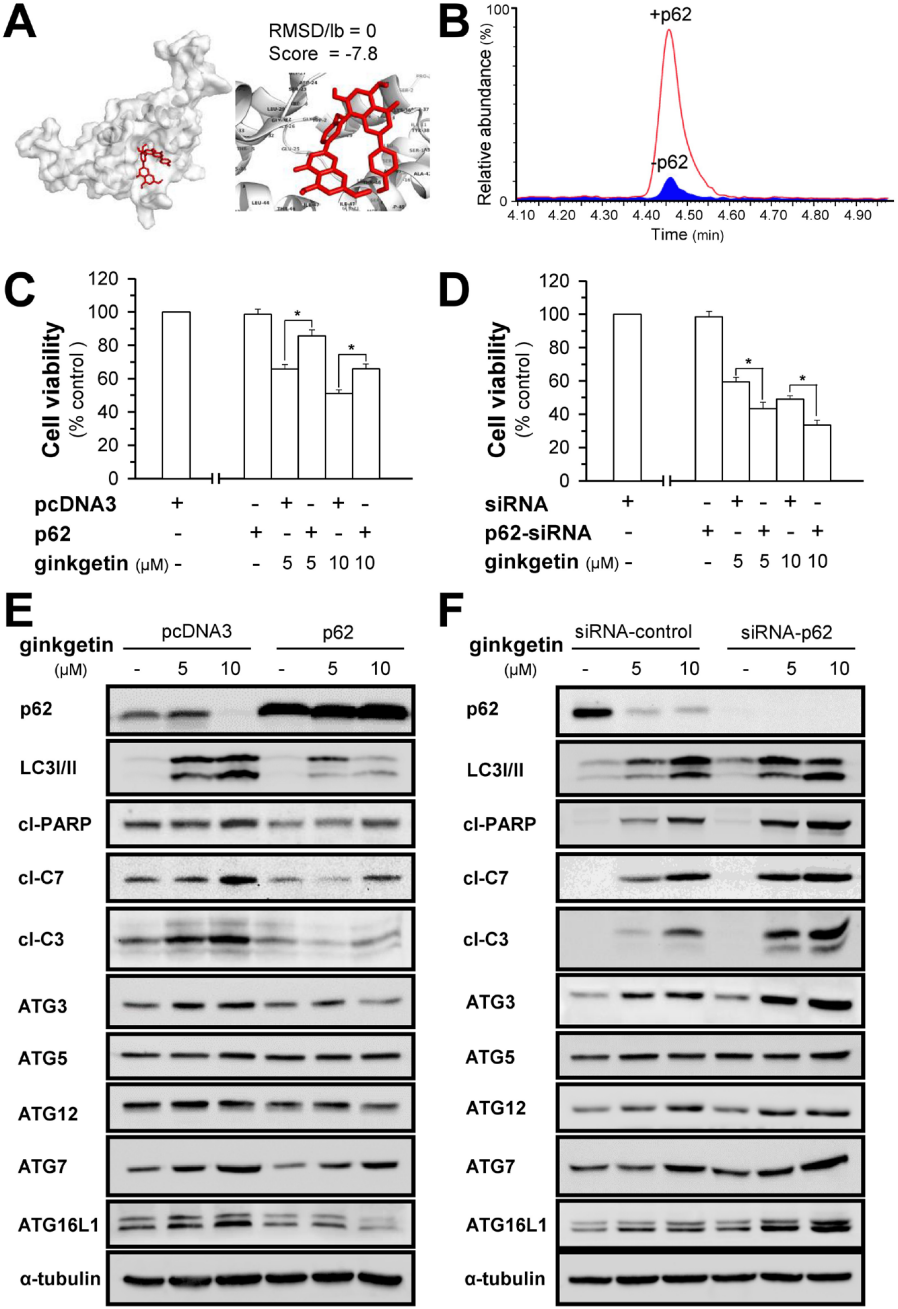
Ginkgetin-induced autophagosome formation is mediated by p62 **(A)** Docking results for ginkgetin and p62. Crystal structure of p62 UBA domain was obtained from “protein data bank”. File formats were reformatted and refined prior to docking approach utilizing AutoDock tools. AutoDock tools and AutoDock Vina was used for docking ginkgetin into p62. The binding structure was visualized by Pymol. The binding score indicated the binding affinity measured in kcal/mol. Root mean square deviation/lower bound (RMSD/lb) values are calculated relative to the best mode (left pannel) and use only movable heavy atoms. RMSD/lb is defined as follows: RMSD/lb (c_1_, c_2_) = max(RMSD'(c_1_, c_2_), RMSD'(c_2_, c_1_)). The proposed highest binding affinity site was shown. Others were shown in Supplementary Figure 3. **(B)** LC trace of ginkgetin mixed with (red line) and without p62 (blue part). 500 μL 0.15 μM ginkgetion solution with or without p62 protein were incubated 1h at 4°C. Then solutions were applied to ultrafiltration tubes to retain p62 to ultrafiltration membrane by three times ultrafiltration. Then, tris buffer was applied to ultrafiltration membrane both with or without p62. ACN was applied to precipitation the p62, the supernatant was applied to Ultra-performance liquid chromatography (UPLC) to analysis the amount of ginkgetin. The amount of ginkgetin was much higher in the presence of p62 after ultrafiltration compared with the control that without p62. **(C)** A549 cells were seeded in 96-well plates (3 ×10^3^ cells/well), allowed to adhere overnight and transfected with pcDNA3 or p62 WT plasmid (100 ng/well) for 4 hours, subsequently treated with ginkgetin for 48 hours. **(E)** As in (C), the protein levels of p62 (∼62 kDa), LC3 I/II (∼14 and ∼16 kDa), cleaved-PARP (cl-PARP; ∼89 kDa), cleaved-caspase 3 (cl-C3; ∼17 and ∼19 kDa) and 7 (cl-C7; ∼20 kDa), ATG3 (∼40 kDa), ATG5 (∼55 kDa), ATG12 (∼55 kDa), ATG7 (∼78 kDa), and ATG16L1 (∼66 and ∼68 kDa), were detected by western blot. Expression of α-tubulin (∼55 kDa) served as a control. **(D** and **F)** Same as in (C) and (E) except siRNA of p62 was used. Values are in percentage of cell growth, in mean ± SEM, *n* = 3.

Disruption of the UBA domain impairs the molecular assembly of p62, consequently disrupting the recognition, sequestration and ingestion of autophagic cargo, leading to malfunction in autophagosome formation [30]. Ginkgetin shows possible binding to the UBA domain of p62 (Figure 4A and Supplementary Figure 3), and the binding affinity was observed when ginkgetin solution was incubated with p62 (Figure 4B and Supplementary Figure 4). The role of p62 in ginkgetin-induced autophagy was further confirmed here by overexpression or knockdown of p62 in cultured A549 and PC9 cells. A cDNA encoding p62 was transfected into the cells, and overexpression of p62 prevented ginkgetin-induced cell death by ∼20% both in A549 (Figure 4C) and PC9 cells (Supplementary Figure 5A). In parallel, p62 overexpression suppressed not only the ginkgetin-induced expression of LC3 I/II but also the apoptotic markers cleaved-PARP, cleaved-caspase 3, and cleaved-caspase 7 in A549 cells (Figure 4E). However, the siRNA-induced knockdown of p62 further increased ginkgetin-induced cell death (Figure 4D). The expression levels of LC3 I/II and of the apoptotic markers were also increased in the p62 knockdown cells (Figure 4F).

The ATG12 and LC3 ubiquitin-like conjugation systems are major processes involved in autophagosome formation. In the ATG ubiquitin-like conjugation system, ATG12 binds to ATG5 via ATG7 and consequently interacts with ATG16L1 to form a dimeric complex to promote autophagosome formation [31]. The overexpression of p62 notably decreased the ginkgetin-induced expression of ATG7 and ATG16L1 (Figure 4E). Although the ATG5 level showed no significant change in the p62-overexpressing cells, ATG12-ATG5 conjugation was notably decreased, as indicated by the protein band of ATG12 at ∼55 kDa (Figure 4E). In a reciprocal manner, the silencing of p62 promoted the expression of ATG7, ATG16L1 and ATG12-ATG5 (Figure 4F). In an LC3 ubiquitin-like system, ATG7, together with the E2-like enzyme ATG 3, could mediate the conjugation of LC3 I and phosphatidylethanolamine to form LC3 II. The increase in LC3 II expression induced by ginkgetin was concomitant with an increase in ATG3 expression. This phenomenon was reversed by p62 overexpression and promoted by p62 knockdown, which indicated that the formation of LC3 II by ginkgetin-activated ATG3 might be mediated by p62 (Figure 4E and 4F). Thus, the administration of ginkgetin in cultured cells could trigger two ubiquitin-like conjugation systems that could be mediated by p62.

### p62 mediates ginkgetin-induced ROS formation

**Figure 5:**
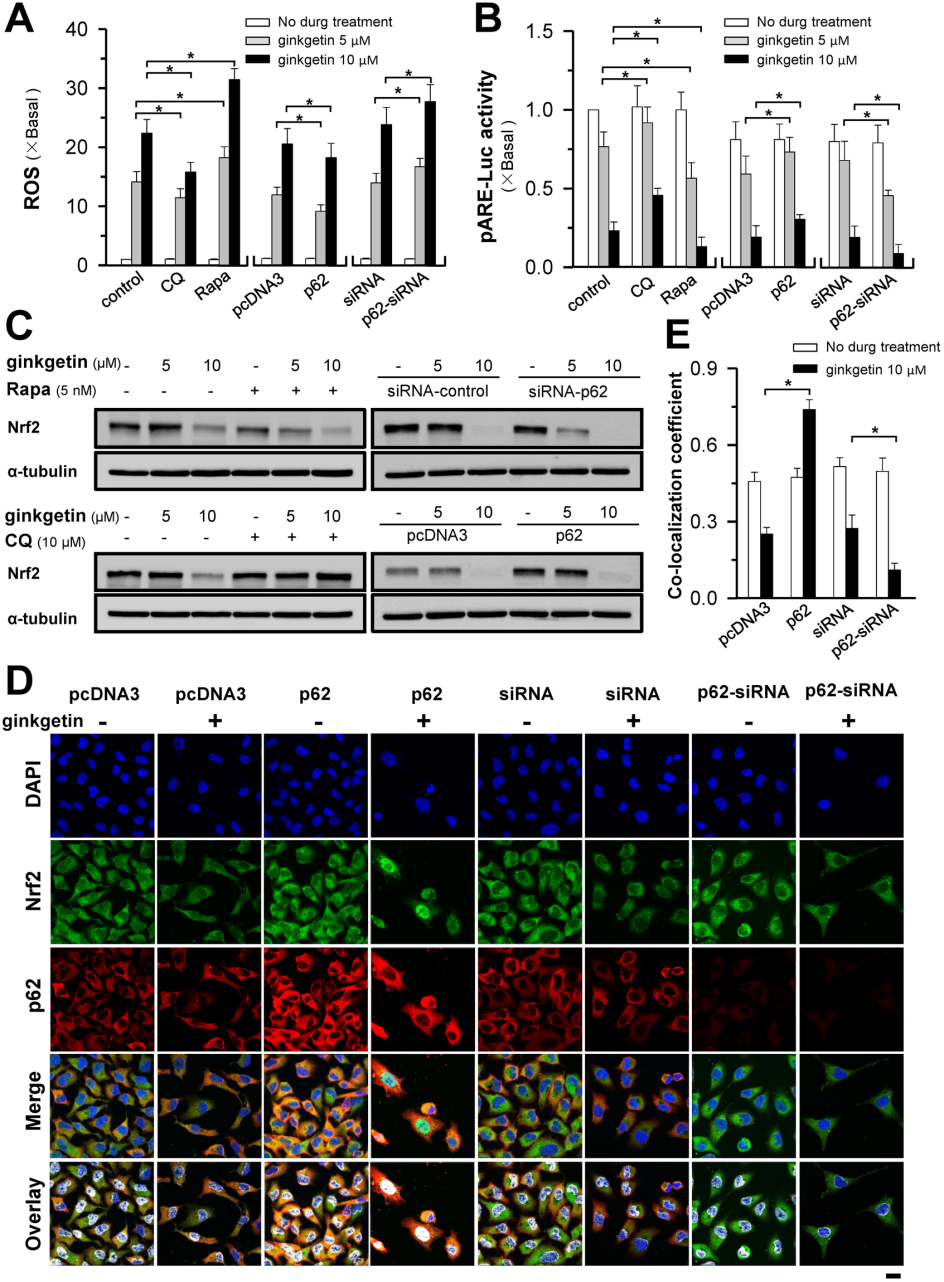
Ginkgetin-reduced Nrf2/ARE activity and ROS formation are mediated by p62 **(A)** A549 cells were seeded in 6-well plates, treated with ginkgetin with or without chloroquine (CQ; 10 μM), or rapamycin (Rapa; 10 nM), or transfected with pcDNA3, or cDNA encoding p62 WT, or siRNA plasmid control, or p62 siRNA, for 4 hours followed by the application of ginkgetin. ROS level was detected by flow cytometry. **(B)** Same as in (A), except pARE-Luc was co-transfected for 4 hours, and pARE-Luc activity was detected by luciferase assay. **(C)** Treatment and transfection were same as in (A), the protein level of Nrf2 (∼100 kDa) was measured by western blot, α-tubulin (∼55 kDa) served as control. **(D)** Transfection was same as in (A), followed by the application of ginkgetin (10 μM). Immunofluorescence staining of A549 cells by antibodies of Nrf2 (green) and p62 (red), and nuclei staining by DAPI (blue). **(E)** Co-localization coefficients from (D), co-localizing pixel for Nrf2 in channel 1 (Ch1) were calculated relative to the total number of pixels for the nuclei (T1) by using Zeiss co-localization coefficient function software. Bar = 20 μm. Representative photos were shown. Values are in fold of change (X Basal) to control (no drug treatment), in mean ± SEM, *n* = 3. ^*^p < 0.05.

Autophagy can promote ROS formation to induce oxidative DNA damage. As mentioned above, we speculated that ROS formation is a consequence of autophagy; thus, the role of p62 in ROS formation was determined here. Administration of ginkgetin in cultured A549 cells robustly increased the formation of ROS, which was suppressed by chloroquine or p62 overexpression and promoted by rapamycin or p62 knockdown (Figure 5A). The consequent event of ROS elevation of DNA damage is the formation of apurinic/apyrimidinic (AP) sites. Here, p62 overexpression reversed the ginkgetin-induced increase in the number of AP sites (Supplementary Figure 6). Similarly, the ginkgetin-suppressed OCR was attenuated by p62 overexpression in A549 cells (Supplementary Figure 7).

Anti-oxidant response element (ARE) is responsible for ROS formation. Ginkgetin robustly decreased the transcriptional activity of ARE (Figure 5B), the outcome of which was inhibited by treatment with chloroquine or p62 overexpression and promoted by rapamycin or siRNA-induced p62 knockdown (Figure 5B). In line with the ROS level and pARE-Luc activity, the expression of Nrf2, an activator of ARE, was regulated in a similar pattern in both A549 and PC9 cell lines (Figure 5C and Supplementary Figure 5B); however, p62 overexpression could not rescue the decrease in Nrf2 with 10 μM ginkgetin treatment. Thus, we further observed the activity of Nrf2 in ginkgetin-treated cells through p62 overexpression and knockdown. The nuclear levels of Nrf2 represent the activity of Nrf2 [32]. Ginkgetin significantly decreased the nuclear translocation of Nrf2, which was reversed by p62 overexpression and promoted by p62 knockdown (Figure 5D and 5E). These results indicated that p62 might play a crucial role in ginkgetin-induced ROS formation.

### Ginkgetin inhibits tumor growth in a xenograft model

**Figure 6:**
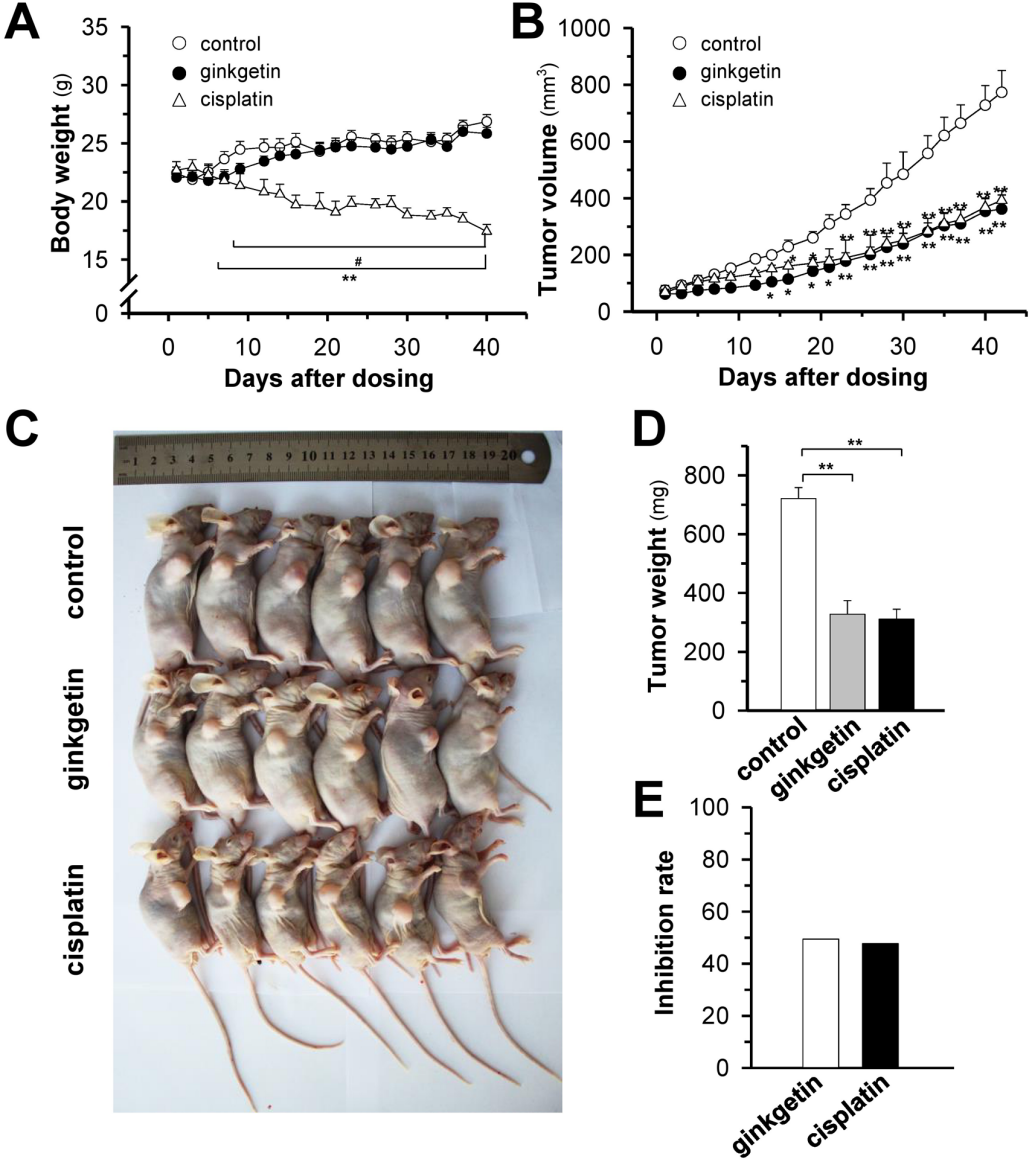
Ginkgetin suppresses tumour growth in A549 cells bearing nude mice Xenograft nude mice model was established by implanting A549 lung cancer cells subcutaneously in the right flank of mice. The tumours were allowed to grow at ∼90 mm^3^. Thereafter, ginkgetin (30 mg/kg, ip), cisplatin (3 mg/kg, ip) were administered. **(A)** The body weight was measured. **(B)** The mean tumour volume in each group after drug treatment. The tumour volume (in cubic millimetres) was calculated using the ellipsoid formula: (D x (d^2^))/2, where “D” represented the large diameter of the tumour, and “d” represented the small diameter. **(C)** Mice bearing tumours were sacrificed at day 42. **(D)** Mean tumour weight in each group at the end of treatment. **(E)** Inhibitory rates of ginkgetin and cisplatin group. The tumour inhibitory rate (IR) was calculated as follows: IR (%) = (1 – TWt/TWc) x 100, where TWt and TWc are the mean tumour weight of treated and control groups, respectively. Values represent mean ± SEM, *n* = 6. ^*^p <0.05 and ^**^p <0.01 vs control group, ^#^ *p* <0.05 vs cisplatin group.

To further explore the anticancer activity of ginkgetin, we established a xenograft nude mouse model. The body weight of mice steadily increased in control and ginkgetin-treated group, and no significant difference was found between these two groups. A robust decrease in body weight (> 20%) was observed after cisplatin treatment (Figure 6A). The tumor size and tumor weight were markedly decreased in groups treated with ginkgetin and cisplatin (Figure 6B-6D). The tumor inhibitory rates of ginkgetin and cisplatin groups were 50% and 48%, respectively (Figure 6E). These results indicated that ginkgetin exhibited promising anticancer effects without obvious toxicity.

### Ginkgetin increases autophagic markers concomitant with decreases mTORC1 and p62/Nrf2 level *in vivo*

**Figure 7:**
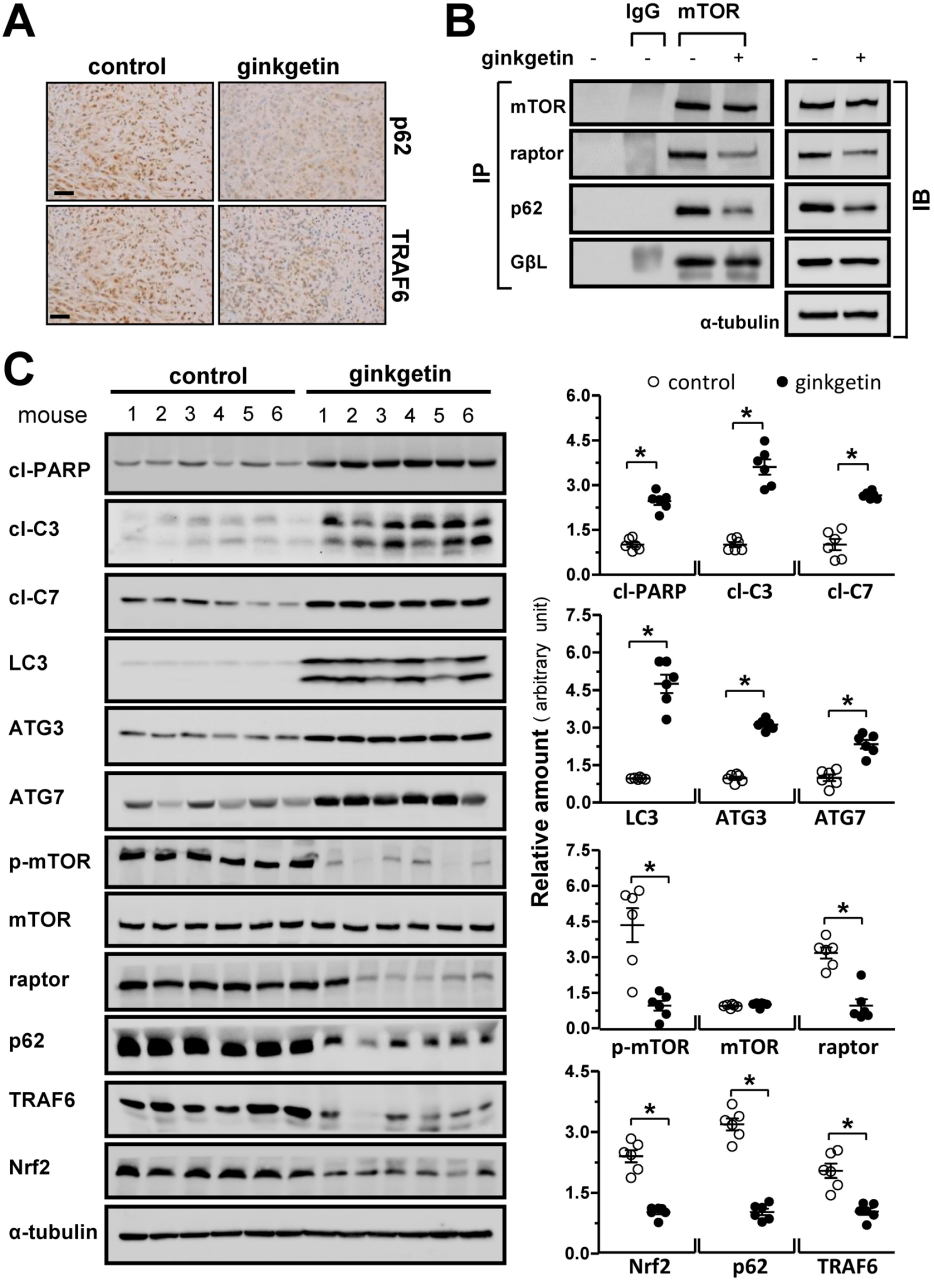
Ginkgetin increases autophagic makers and decreases mTOC1 and p62/Nrf2 level *in vivo* **(A)** Immuno-histochemical observation on the expressions of p62 and TRAF6 in tumour, brown particles represent the antibody staining. Bar = 50 μm. **(B)** Tumour samples of control and ginkgetin-treated groups were lysed, andimmunoprecipitation with the antibody of mTOR (1 μg) and protein G agarose, IgG (1 μg) was added to lysis of untreated group together with protein G agarose as control, and immunoblotting with specific antibodies (left panel). Total cell lysates were probed with the antibodies (right panel). Amounts of mTOR (∼289 kDa), p62 (∼62 kDa), raptor (∼150 kDa), GβL (∼37 kDa) were detected. Expression of α-tubulin (∼55 kDa) served as a control. **(C)** Expression of cleaved-PARP (cl-PARP; ∼89 kDa), cleaved-caspase 3 (cl-C3; ∼17 and ∼19 kDa) and 7 (cl-C7; ∼20 kDa), LC3 I/II (∼14 and ∼16 kDa), ATG3 (∼40 kDa), ATG7 (∼78 kDa), p-mTOR (∼289 kDa), mTOR (∼289 kDa), raptor (∼150 kDa), p62 (∼62 kDa), TRAF6 (∼58 kDa) and Nrf2 (∼100 kDa) in tumours of ginkgetin-treated mice (left panel). Expression of α-tubulin (∼55 kDa) served as a control. Quantitation of protein expression is shown in right panel. ^*^p <0.05 vs control group. *n* = 6.

Next, the tumors from post-mortem mice were analyzed. Immunohistochemical analysis of the tumors revealed that the expression levels of p62 and TRAF6 were markedly reduced in the ginkgetin group compared to those in the control group, which was confirmed by western blot (Figure 7A and 7C). The decline in p62/Nrf2 levels was concomitant with increases in the levels of the apoptotic markers cleaved-PARP, cleaved-caspase 3 and cleaved-caspase 7 and of the autophagosome formation and lysosome acidification markers LC3, ATG3 and ATG7 (Figure 7C). The phosphorylation of mTOR at the 2448 site was significantly decreased in the tumors of ginkgetin-treated mice. In addition, the expression of raptor in tumor and the binding of raptor and p62 to mTOR were notably decreased in the tumors of mice treated with ginkgetin (Figure 7B). These results indicated that ginkgetin-treated mice showed decreased activity of p62-mTORC1 and p62/Nrf2 accompanied by increased levels of autophagic markers.

## DISCUSSION

Three NSCLC cell lines and a xenograft tumor mouse model were highly responsive to ginkgetin challenge, as observed by their increased cell death. The IC50 values in all cell lines were well below those of cisplatin. In addition, the toxicity triggered by ginkgetin was much lower than that of cisplatin, as revealed in the body weights observed in the nude mouse xenograft model. This is the first report of the novel discovery of the anti-cancer effect of ginkgetin in NSCLC.

Autophagic cell death could be a major cause of cell death in ginkgetin-treated A549 cells. This notion is strongly supported by the following experimental outcomes. First, the ginkgetin-treated A549 cells exhibited morphologic manifestations of autophagy rather than of apoptosis, i.e., greater appearance of extensive autophagic vacuoles in the cytoplasm rather than chromosome condensation in the nucleus. Second, according to the recommendation of the Nomenclature Committee on Cell Death, if cell death can be blocked by pharmacological inhibition or genetic targeting, it could be considered ‘autophagic cell death’ [33]. Here, the autophagy inhibitors chloroquine and 3-methyladenine, not the apoptosis inhibitor Q-VD-OPh, reversed ginkgetin-induced cell death. In addition, p62 overexpression could mimic the decrease in autophagy and could partially reverse ginkgetin-induced cell death. Third, the level of apoptosis was positively related to autophagy, i.e., apoptotic markers decreased when autophagy was blocked by pharmacological and genetic manipulation. Thus, autophagy could be the inducer of ginkgetin-induced cell death, and this induction might act through lysosome acidification and autophagosome formation. Supporting this notion, E64D/PA, which targets the late stage of autophagy, did not affect cell survival.

Here, the key roles of p62 in ginkgetin-induced autophagy were illustrated. (i) Ginkgetin exhibited binding affinity to p62 as demonstrated by ultrafiltration-based LC-MS binding assay. (ii) Ginkgetin fully blocked the expression of p62, and ginkgetin-induced toxicity was blocked by p62 overexpression. (iii) Formation of p62-mTOR complex was negatively related to ginkgetin-induced lysosome acidification. (iv) Ginkgetin-induced autophagosome formation was reversed by p62 overexpression. (iv) The modulation of p62 expression regulated the ginkgetin-induced decrease in Nrf2 activity and increase in ROS formation.

The impairment of autolysosome formation includes disruptions of lysosome acidification, autophagosome-lysosome fusion and autophagosome formation [34]. p62 interacts with mTORC1, forming the p62-mTORC1 complex, and this complex formation is negatively related to lysosome acidification and autophagosome-lysosome fusion [26, 35, 36]. In addition, p62 interacts with TRAF6 to promote its oligomerization, which contributes to the formation of p62-mTORC1 [27]. The elevation of p62 by chemical approaches in ginkgetin-treated cells occurred concomitant with autophagy inhibition, TRAF6 elevation and p62-mTORC1 complex formation, which consequently reduced ginkgetin-induced lysosome acidification. In parallel, the attenuation of the expression of ATG7, a key protein regulating the fusion of autophagosomes and lysosomes, was observed in ginkgetin-treated A549 cells after p62 overexpression by both chemical and genetic approaches. As confirmation of this phenomenon, p62 knockdown through chemical and genetic means exhibited reciprocal effects. Therefore, ginkgetin-induced suppression of p62-mTORC1 formation and elevation of ATG7 expression were important for lysosome acidification.

The amount of p62 was negatively regulated not only during lysosome fusion and acidification but also during autophagosome formation. The silencing of p62 could promote autophagosome formation in several cancer cell lines [37]. In addition, the p62-mTORC1 complex could be localized in the Golgi, which enables mTORC1 activation and hence indirectly inhibits autophagosome formation [38]. As shown in our study, the degree of autophagy induced by ginkgetin in A549 cells was negatively related to the expression of p62. Autophagosome formation mainly undergoes four steps: initiation via the ATG1 complex, nucleation and assembly to form a double-membrane phagophore via the ATG2-ATG9 complex, membrane expansion with the assistance of conjugation systems, and vesicle completion via ATG7. ATG proteins are the major components in ATG12 ubiquitin-like conjugation and LC3 ubiquitin-like conjugation systems. For instance, autophagy deficiency was identified in cells lacking ATG proteins involved in these two conjugation systems (i.e., ATG3, ATG5, ATG7 and ATG16L1) [39–42]. Ginkgetin promoted the expression of ATG3, ATG7, ATG16L1, and the ATG12-ATG5 complex but had no effect on ATG5. The results for ATG5 and ATG12-ATG5 were consistent with a previous study, which showed that the expression levels of ATG12-ATG5 and free ATG 12 were increased after the starvation of lung epithelial BEAS-2B cells; however, no obvious change in ATG 5 was observed [43]. The increase in the ATG12-ATG5 complex may be due to either the increased amount of free AGT12 or the interaction of ATG12 and ATG5 through covalent and non-covalent linkages [44]. Thus, the ginkgetin-induced increase in ATG12-ATG5 may be due to the change in covalent or non-covalent linkages between ATG12 and ATG5. Here, the overexpression or knockdown of p62 modulated the ginkgetin-induced elongation of phagophores in cultured A549 cells through ATG proteins (e.g., ATG 3, ATG 7, ATG16L1, and the ATG12-ATG5 complex). These outcomes indicated that p62 may function in ginkgetin-induced autophagosome formation through ATG12 ubiquitin-like and LC3 ubiquitin-like conjugation systems and consequently disrupt autolysosome formation. Furthermore, p62-mTORC1 complex could lead to decrease in autolysosome formation through directly or indirectly inhibiting lysosome acidification and autophagosome formation [34, 37, 38, 45], and ginkgetin-induced suppression of the p62-mTORC1 complex was observed. Thus, p62 may have a crucial role in ginkgetin-induced autolysosome formation.

The interaction of p62 with Nrf2 could result in the direct binding of activated Nrf2 to the ARE of DNA regulatory elements in anti-oxidative genes to reduce ROS formation [46–48]. Ginkgetin-induced autophagy could be a cause of ROS formation. First, the ROS scavenger N-acetyl-L-cysteine was not able to rescue ginkgetin-induced cell death. Second, the suppression of autophagy by chloroquine or overexpression of p62 reversed ginkgetin-induced cell death and induced a decrease in ROS. The ROS formation triggered by ginkgetin was negatively related to p62/Nrf2/ARE activity. Although the amount of Nrf2 with 10 μM ginkgetin treatment was not rescued by p62 overexpression, the activity was largely rescued by p62 overexpression. Furthermore, p62 was responsible for the ginkgetin-induced OCR decrease and AP site increase. Thus, p62 might serve as an upstream effector of ginkgetin-induced ROS formation and be critical in the redox setting during the process of ginkgetin-induced autophagic cell death.

Ginkgetin, derived from *G. biloba* leaves (<0.02% by dry weight) [49], has been shown to have anti-cancer activity in prostate cancer cells at micromolar concentrations [50]. In renal cell carcinoma and osteosarcoma, ginkgetin induced cell apoptosis through JAK2/STAT3 signaling [51, 52]. We have screened several potential targets for ginkgetin, including Beclin1 and ATG7, and p62 is believed to be the most functional target (Supplementary Figure 8). However, which residue of p62 is responsible for ginkgetin binding and how this process modulates its function need to be further investigated. In addition, an extract from *G. biloba* leaves was demonstrated to possess anticancer effects in a dose-dependent manner (Supplementary Figure 9). *G. biloba* extract-treatment of sarcoma 108 (S180)-bearing mice significantly reduced tumor weight [53]. Despite the belief that ginkgetin originates from *G. biloba*, this chemical has also been reported in *Taxus chinensis*, *Selaginella doederleinii*, and *Gaultheria yunnanensis*. In particular, *T. chinensis*, the source of Taxol, is responsible for the production of approximately 0.3% of available ginkgetin [54]. These lines of evidence strongly support a novel function of ginkgetin in anticancer activity.

Natural products are receiving considerable attention for the prevention and treatment of cancer because of their promising efficacy and low toxicity. Recent studies have suggested that autophagy may serve as a novel therapeutic target for cancer. For instance, isocryptotanshinone from the roots of *Salvia miltiorrhiza* (red saga) induced autophagic cell death in A549 cells [55], and salvianolic acid B triggered autophagy in cultured HCT116 and HT29 cells [56]. Our study is the first to demonstrate the anticancer activity of ginkgetin in NSCLC with strong autophagy induction, and a similar anticancer effect is seen in the orthodox chemotherapeutic drug cisplatin without obvious toxicity in a nude mouse xenograft model. Thus, ginkgetin is proposed to be a potential compound for novel anticancer drug development for NSCLC.

## MATERIALS AND METHODS

### Reagents and antibodies

A FITC-labeled Annexin V Apoptosis Detection Kit was obtained from BD Biosciences (San Jose, CA). JC-1, cisplatin, and DCFH-DA were purchased from Sigma-Aldrich (St. Louis, MO). Cisplatin, for use in the animal study, was purchased from Shandong Qilu Pharmaceutical (Shandong, China). Ginkgetin (>98% purity) was purchased from Chengdu Must Bio-technology Ltd. (Chengdu, China). The culture medium was obtained from Invitrogen Technologies (Carlsbad, CA). The antibodies were obtained from the following sources: p62, TRAF6, and Nrf2 were obtained from Abcam (Cambridge, UK); α-tubulin from Sigma-Aldrich; and LC3 I/II, c-JUN, p-AKT, cleaved-PARP, cleaved-caspase 3, cleaved-caspase 7, ATG3, ATG5, ATG7, ATG12, ATG16L1, p-mTOR, mTOR, raptor, GβL, horseradish peroxidase (HRP)-conjugated goat anti-rabbit antibody, HRP-conjugated goat anti-mouse antibody, Alexa Fluor 555-conjugated goat anti-mouse antibody, and Alexa Fluor 488-conjugated goat anti-rabbit antibody from Cell Signaling Technology (Danvers, MA). p62 siRNA was obtained from Cell Signaling. The p62 plasmid was kindly gifted by Dr. Alexander Shneider from CureLab Oncology, Inc. (Needham, MA), and the pARE-Luc plasmid was obtained from Promega Corporation (Madison, WI). The recombinant p62/SQSTM1 protein was purchased from Novus Biologicals (Littleton, CO). A549, PC9, and NCIH460 cell lines were obtained from American Type Culture Collection (ATCC, Manassas, VA).

### Cell viability assay

MTT assay was performed as previously described [57]. In brief, cells were plated in 96-well plates at 3000 cells per well and subsequently treated with various concentrations of cisplatin and ginkgetin for 24, 48, and 72 h. MTT was added, and the formazan crystals that formed were dissolved in dimethylsulfoxide. Spectrophotometric absorbance at 570 nm was determined.

### Transmission electron microscopy

Cultured A549 cells were treated with ginkgetin at 10 μM for 24 h. Cells were harvested, washed with PBS and then fixed in 2.5% glutaraldehyde for 4 h at 4°C. The samples were washed with PBS and then treated with 1% osmium tetroxide for 1 h. After the samples were washed, they were dehydrated in a graded series of ethanol (50%, 70%, and 90%) and acetone and then embedded in durcupan resin. Thin sections (120 μM) were post-stained with uranyl acetate and lead citrate before examination under a Philips TECNAI 10 transmission electronic microscope (TEM) [58].

### Measurement of apoptosis, intracellular ROS, and MMP

The apoptosis assay was conducted using an Annexin V-FITC/PI Apoptosis Detection Kit in accordance with the manufacturer's instructions. The samples were analyzed using a FACSAria equipped with CellQuest Software (BD Biosciences) [59]. The levels of ROS and MMP were measured as previously described. In brief, cells were washed, collected, and stained with DCFH-DA or JC-1, and then, the ROS and MMP were detected by flow cytometry [60].

### Western blot analysis

Western blot was performed as previously described [57]. In brief, the cells and tissue samples were lysed, and their total protein concentrations were measured using the Bradford method. Equal amounts of proteins were separated by SDS-PAGE and then transferred to nitrocellulose membranes. The membranes were blocked and then probed with the indicated primary antibodies. The blots were rinsed and then incubated with secondary antibodies. Reactive bands were visualized using ECL (Thermo Scientific) and then calibrated by the Chemidoc Imaging System (Bio-Rad; Hercules, CA).

### Immunoprecipitation

The cells or tumors were rinsed once with PBS and then lysed in CHAPS buffer (10 mM HEPES (pH 7.4), 150 mM NaCl, 1 mM EDTA, 1 mM EGTA, 0.4% CHAPS, 3 mM benzamidine, 20 μg/mL leupeptin, 20 μg/mL aprotinin, 20 mM NaF, 1.5 mM Na_3_VO_4_, and 50 mM β-glycerophosphate). The soluble fractions were isolated by centrifugation at 13,000 rpm for 30 min. Primary antibody was added, and IgG was added to the control sample, followed by overnight incubation at 4°C with rotation. Then, 50 μL of homogeneous protein G-agarose suspension (Roche) was added to the mixture and then incubated overnight at 4°C. The precipitated products were washed and denatured. Cell extracts or immunoprecipitated proteins were detected by western blot [61].

### Lysosome acidification assay

Approximately 1 × 10^5^ cells were seeded in cover glasses (Marienfeld Superior, Germany, thickness No.1, ø = 24 mm), incubated overnight, and then treated with ginkgetin in the absence or presence of either chloroquine or rapamycin for 12 h. The cells were stained with 100 nM LysoTracker® Red DND-99 (Thermo Fisher, MA) for 1 h and then incubated with 1 mg mL^−1^ Hoechst 33258 (Yeasen, Shanghai, China) for 20 min. After staining, the cover glass was washed and fixed (Aireka, FL). The cells were viewed under a Zeiss Laser Scanning Confocal Microscope (LSM7 DUO).

### Luciferase activity

The vector pGL4.37 [*luc2P*/ARE/Hygro] carrying four repeats of anti-oxidant response elements (ARE: 5′-TAG CTT GGA AAT GAC ATT GCT AAT GCT GCT GAG TCA ACT TT-3′) and a luciferase reporter gene *luc2P* (*Photinus pyralis*) was named pARE-Luc. Cultured A549 cells were co-transfected with pARE-Luc and p62 WT plasmid or siRNA for 4 h by jetPRIME® (Invitrogen) in accordance with the manufacturer's instructions and then treated with ginkgetin for an additional 24 h. Chloroquine or rapamycin was cotreated with ginkgetin for 24 h. Luciferase assay was conducted as previously described [62]. In brief, the cells were washed and lysed. The supernatant was collected and then analyzed using a commercial kit (Thermo Fisher Scientific, Waltham, MA).

### Immunofluorescence

Immunofluorescence detection was performed as previously described [57]. In brief, the cells were washed and fixed. After blocking, primary antibodies were applied, washed, and then probed with Alexa Fluor®-conjugated secondary antibodies and DAPI. Images were taken using a Zeiss Laser Scanning Confocal Microscope (LSM7 DUO). The coefficients of colocalization were calculated using the Zeiss colocalization function software.

### Measurement of OCR

Seahorse XFp Extracellular Flux Analyzer (Seahorse Biosciences, North Billerica, MA) was used to obtain real-time measurements of the OCR in cells. Cell preparation and analysis assay were performed using a Mito Stress Test Kit (Seahorse Biosciences) in accordance with the manufacturer's instructions. In brief, the cells were seeded in quintuplets in eight-well cartridges (1.2 × 10^5^ cells/well). The cartridge with cells was placed into a Seahorse Analyzer incubator unit for 60 min before running a program for equilibration. The basal rates of oxygen consumption were measured three times during the first 15 min, and oligomycin (50 μM), carbonyl cyanide-*p*-trifluoromethoxy-phenylhydrazone (FCCP) (50 μM), and a mixture of anti-mycin A and rotenone (25 μM) were subsequently added to the cells, followed by three measurements for each statement [63].

### Ultrafiltration-based affinity assay

In total, 500 μL 0.15 μM ginkgetin solution with or without p62 protein was incubated 1 hour at 4°C. Then, solutions were applied to ultrafiltration tubes to retain p62 on the ultrafiltration membrane by three rounds of ultrafiltration. Then, tris buffer was applied to the ultrafiltration membrane. ACN was applied to precipitate the p62; the supernatant was subjected to ultra-performance liquid chromatography (UPLC) to analyze the amount of ginkgetin. Ultra-performance liquid chromatography (UPLC) was performed using a Waters ACQUITY UPLC system (Waters, Milford, MA, USA) with an ACQUITY UPLC BEH C18 column (2.1 × 50 mm, 1.7 μm). The solvents used were as follows: A, 0.1% diluted aqueous formic acid; B, 0.1% formic acid in acetonitrile (ACN). The gradient conditions for LC-MS were as follows: 0–7 min, 2–98% B; 7–8 min, 98–2% B; and 8–10 min, 2% B. The column and sample temperatures were maintained at 35°C and room temperature, respectively. Mass spectrometric detection was coupled with UPLC and performed using a Synapt™ quadrupole time-of-flight (Q-TOF) High-Definition Mass Spectrometer (Waters, Milford, MA, USA) equipped with an electrospray ionization (ESI) source operating in positive ionization mode. The optimized mass spectrometric parameters were determined as follows: capillary voltage, 2.5 kV; sample cone, 25 V; extraction cone, 4.0 V; source temperature, 120°C; and desolvation temperature, 350°C. Nitrogen was used as a desolvation and a cone gas at a flow rate of 600 and 50 L h^−1^, respectively. Argon was used as a collision gas. A lock mass of leucine-enkephalin at a concentration of 200 pg mL^−1^ in 50% ACN-water solution (including 0.1% formic acid) was employed as an external reference to generate a [M + H]^+^ ion in positive mode at m/z 556.2771 via a lock spray interface at a flow rate of 5 mL min^−1^ to acquire accurate mass during the analysis. The sample was scanned in full-scan mode from m/z 80 to 800 in 1 s scan intervals [64].

### Animal xenograft model

A xenograft nude mouse model was established as previously described [57]. In brief, A549 tumor pieces were implanted into the left flank of 5-week-old (18-22 g) male BALB/C nu/nu nude mice that were obtained from Shanghai SLAC Laboratory Animal Company (Shanghai, China). The nude mice were maintained in pathogen-free conditions at 22°C ± 2°C at 70% relative humidity and under a 12-hour light/dark cycle. On day 20 post-tumor implantation, the mice were randomized into three groups (*n*=6) according to their tumor volume so that all the groups had a similar starting mean tumor volume. Cisplatin (3 mg kg^−1^) was administered two times per week by intraperitoneal injection, whereas the ginkgetin solution (2% DMSO, 6% cremophor EL, 92% NaCl, and 30 mg kg^−1^) was administered intragastrically once per day. For preparation of ginkgetin formulation, ginkgetin powder was dissolved in DMSO (2% of total volume) and sonicated for 30 min until the solution became clear. Then, we vortexed the solution while slowly adding cremophor EL (6% of total volume) and NaCl (92% of total volume). Tumor size was measured three times per week. The nude mice were sacrificed by cervical dislocation after treatment, and the tumor weights were recorded. All animal experiments were performed in accordance with the National Institutes of Health Guide for the Care and Use of Laboratory Animals and were approved by Hangzhou Hibio Experimental Animal Ethics Committee (Permit Number: HB201511013) under the guidelines of the “Principles of Laboratory Animal Care” (NIH publication No. 80-23, revised 1996), Institutional Animal Care and Use Committees protocol (HBFM3.68-2015) and ARRIVE guidelines [65, 66].

### Statistical analyses

All experiments were blinded and randomized. Data are expressed as the mean ± standard error of the mean (SEM). Statistical comparisons were performed using one-way analysis of variance (ANOVA) followed by a Bonferroni multiple comparisons test using SPSS 16.0 software (Chicago, IL, USA). Unpaired t-test was used when comparing two groups. Statistical significance was achieved at ^*^p < 0.05, ^**^p < 0.01, and ^***^p < 0.001. Post-tests were conducted when F achieved *p* < 0.05 and no significant variance in homogeneity was observed.

## SUPPLEMENTARY MATERIALS FIGURES


