# Supplementary material for: Ginkgetin induces autophagic cell death through p62/SQSTM1-mediated autolysosome formation and redox setting in non-small cell lung cancer
Source: Oncotarget. 2017 Oct 16;8(54):93131–48. doi: 10.18632/oncotarget.21862 (PMC5696249; doi:10.18632/oncotarget.21862)
Supplement: Supplementary file 1 [file oncotarget-08-93131-s001.pdf]

Ginkgetin induces autophagic cell death through p62/SQSTM1-mediated autolysosome formation and redox setting in non-small cell lung cancer

SUPPLEMENTARY MATERIALS

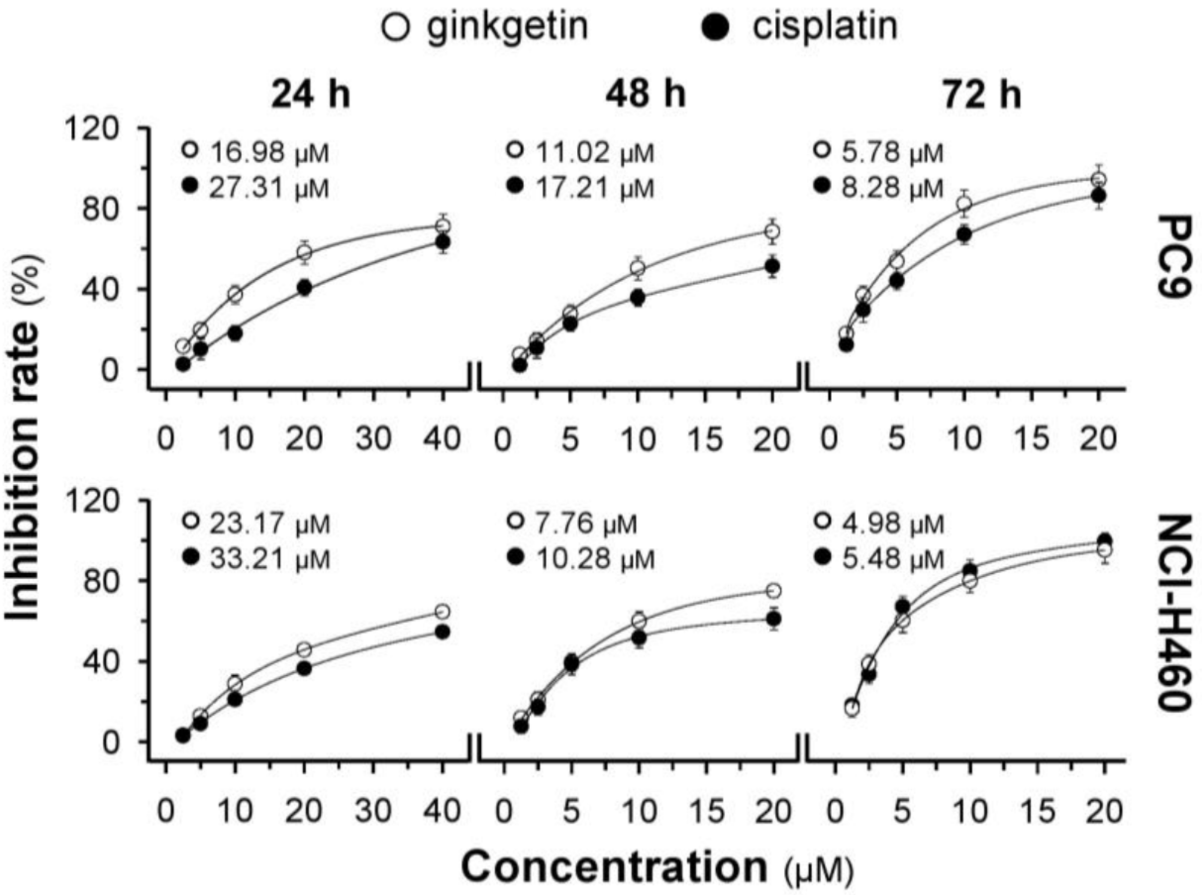

Supplementary Figure 1: Ginkgetin induces cell death in non-small cell lung cancer. Cytotoxicity of ginkgetin was observed in NSCLC cell lines: PC9 and NCIH460. Cells were seeded in 96-well plates (3 × 10^3 cells/well) and subsequently were treated with ginkgetin or cisplatin. MTT assay was employed to reveal the toxicity of ginkgetin. The IC_{50} in different scenarios are shown. Values are in percentage of cell growth inhibition. Each point represents mean ± SEM, *n* = 3.

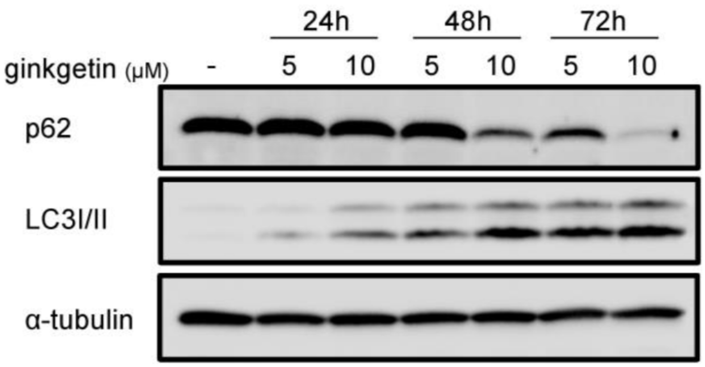

Supplementary Figure 2: Ginkgetin induces p62 reduction and LC3 elevation. A549 cells were treated with ginkgetin at different doses for 24, 48 and 72 hours. The protein expressions of LC3 I/II (~14 and ~16 kDa) and p62 (~62 kDa), were determined by western blotting. Expression of a-tubulin (~55 kDa) served as a control.

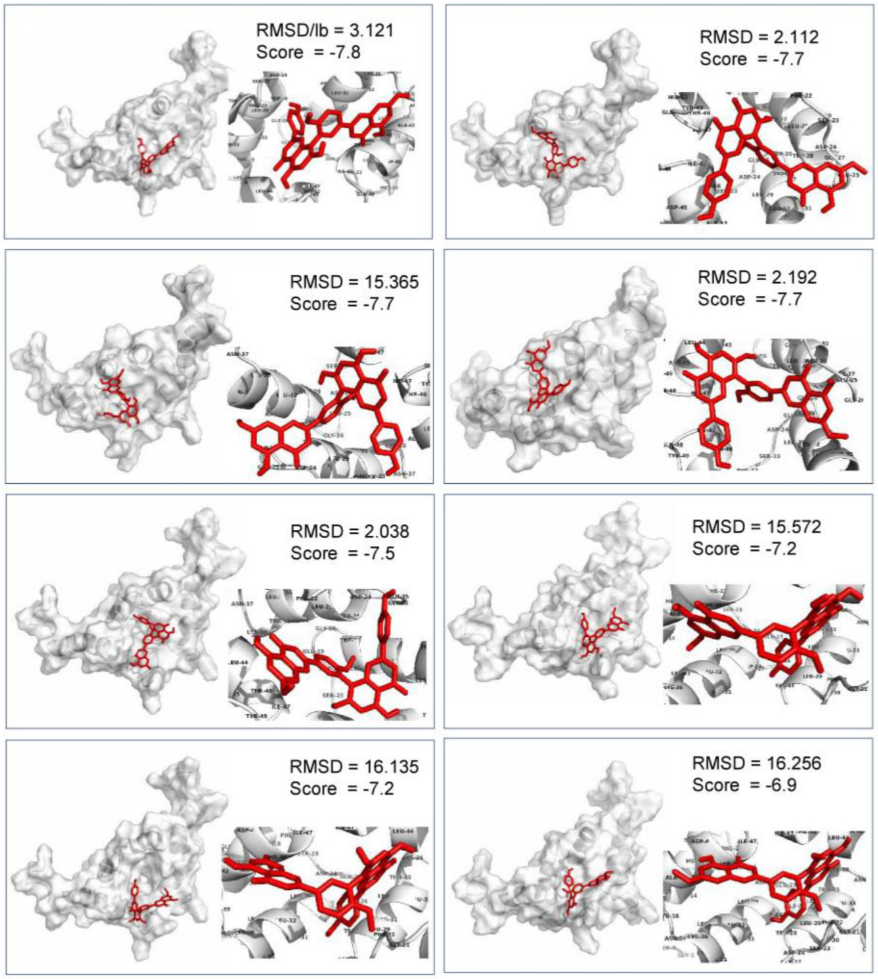

Supplementary Figure 3: Docking results for ginkgetin and p62. Crystal structure of p62 UBA domain was obtained from "protein data bank". File formats were reformatted and refined prior to docking approach utilizing AutoDock tools. AutoDock tools and AutoDock Vina was used for docking ginkgetin into p62. The binding structure was visualized by Pymol. The binding score indicated the binding affinity measured in kcal/mol. Root mean square deviation/lower bound (RMSD/lb) values were calculated relative to the best mode (left pannel) and used only movable heavy atoms. RMSD/lb was defined as follows: RMSD/lb $(c_1, c_2) = \max(\text{RMSD}'(c_1, c_2), \text{RMSD}'(c_2, c_1))$.

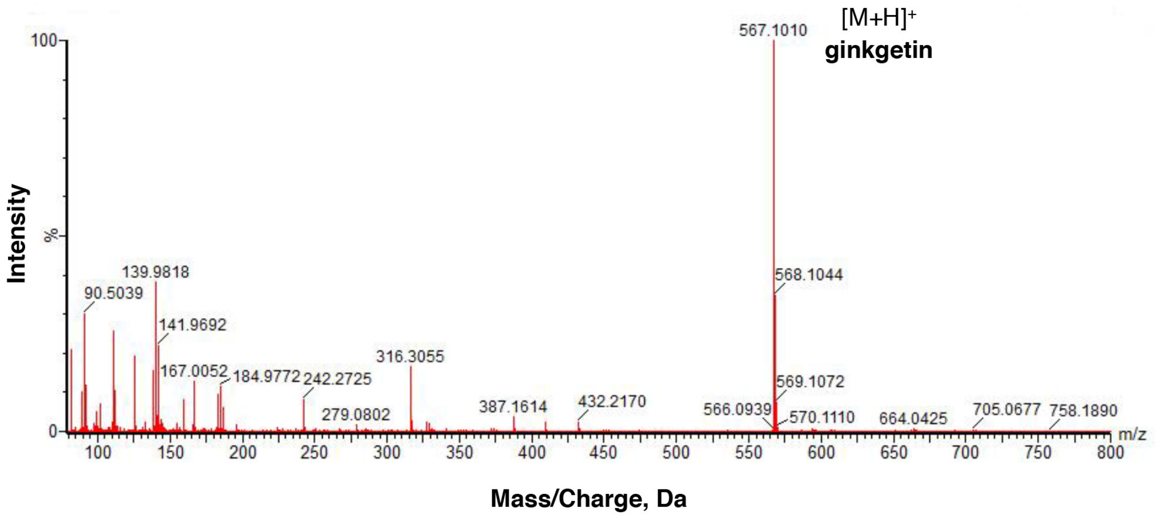

Supplementary Figure 4: Mass chromatogram of ginkgetin. Ultra-performance liquid chromatography (UPLC) was performed using a Waters ACQUITY UPLC system (Waters, Milford, MA) with an ACQUITY UPLC BEH C18 column (2.1 × 50 mm, 1.7 µm). The solvents used were as follows: A, 0.1% diluted aqueous formic acid; and B, 0.1% formic acid in acetonitrile (ACN). The gradient conditions for LC-MS were as follows: 0–7 min, 2–98%B; 7–8 min, 98–2% B; and 8–10 min, 2% B, and the column and sample temperatures were maintained at 35°C and room temperature, respectively. Mass spectrometric detection was coupled with UPLC and performed using a Synapt™ quadrupole time-of-flight (Q-TOF). High-Definition Mass Spectrometer (Waters, Milford, MA) equipped with an electrospray ionization (ESI) source operating in positive ionization mode. The optimized mass spectrometric parameters were determined as follows: capillary voltage, 2.5kV; sample cone, 25 V; extraction cone, 4.0 V; source temperature, 120 °C; and desolvation temperature, 350 °C. Nitrogen was used as a desolvation and a cone gas at a flow rate of 600 and 50 L h^{-1}, respectively. Argon was used as a collision gas. A lock mass of leucine-enkephalin at a concentration of 200 pg mL^{-1} in 50% ACN–water solution (including 0.1% formic acid) was employed as an external reference to generate a $[M + H]^+$ ion in positive mode at m/z 556.2771 via a lock spray interface at a flow rate of 5 mL min^{-1} to acquire accurate mass during the analysis. The sample was scanned in full-scan mode from m/z 80 to 800 in 1 s scan intervals.

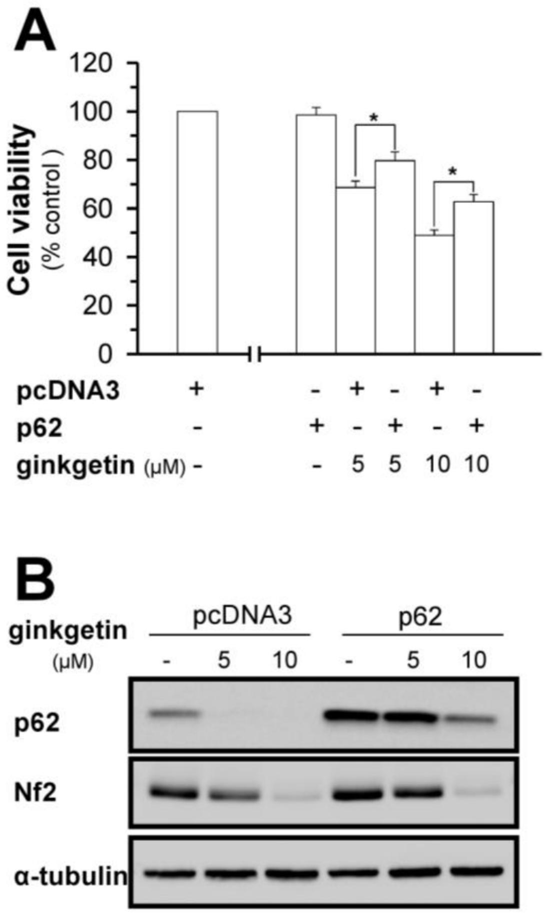

Supplementary Figure 5: Overexpress p62 reduces ginkgetin induced toxicity and increase Nrf2 level in PC9 cell line. **(A)** PC9 cells were seeded in 96-well plates (3×10^3 cells/well), allowed to adhere overnight and transfected with pcDNA3 or p62 WT plasmid (10 ng/well) for 4 hours, subsequently treated with ginkgetin for 48 hours. Values are in percentage of cell survival rate. Each point represents mean ± SEM, $n = 3$. **(B)** A549 cells were transfected with pcDNA3, or cDNA encoding p62 WT for 4 hours followed by the application of ginkgetin. the protein levels of p62 (~62 kDa) and Nrf2 (~100 kDa) were measured by western blot, α-tubulin (~55 kDa) served as control.

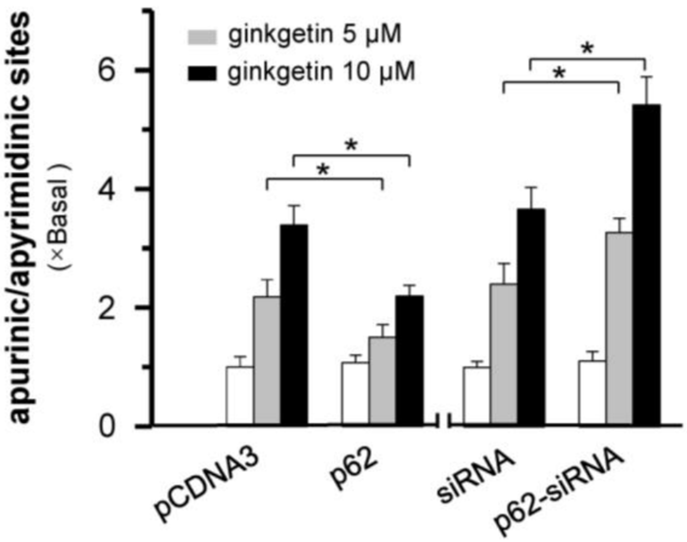

Supplementary Figure 6: p62 decreases ginkgetin-induced apurinic/apyrimidinic sites elevation. A549 cells were transfected with pcDNA3, or cDNA encoding p62 WT, or siRNA plasmid control, or p62 siRNA, for 4 hours followed by the application of ginkgetin. Genomic DNA was extracted. The number of apurinic/apyrimidinic sites in the DNA was detected by DNA Damage Quantification Colorimetric Kit (BioVision, Milpitas, CA) following the manufacturer's instructions. Values are relative amount in fold of change (X Basal) to control (no drug treatment). Results are expressed as the mean ± SEM, $n = 3$. $^{*}p < 0.05$.

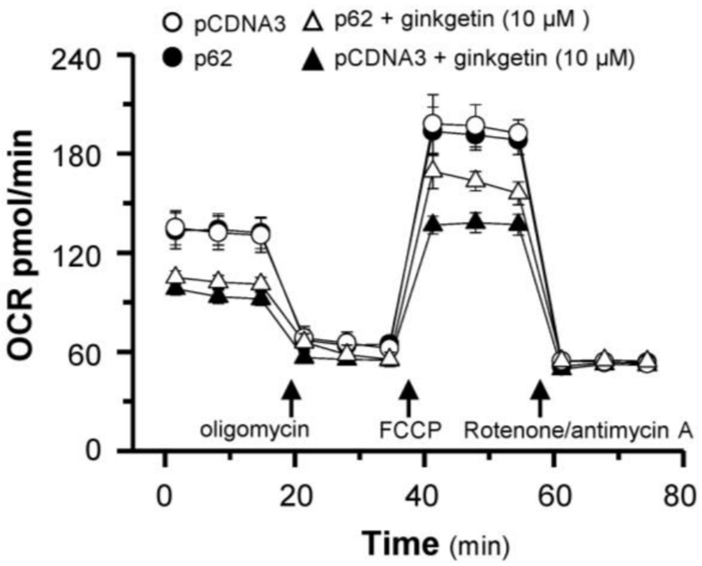

Supplementary Figure 7: p62 contributes ginkgetin-induced OCR reduction. Cultured A549 cells were transfected with pcDNA3, or cDNA encoding p62 WT for 4 hours followed by the application of ginkgetin (10 μM) for 6 hours. Oxygen consumption rate (OCR) measurements were performed with Mito Stress Test Kit and were analysed by Seahorse Instrument. Results are expressed as mean ± SEM, $n = 3$.

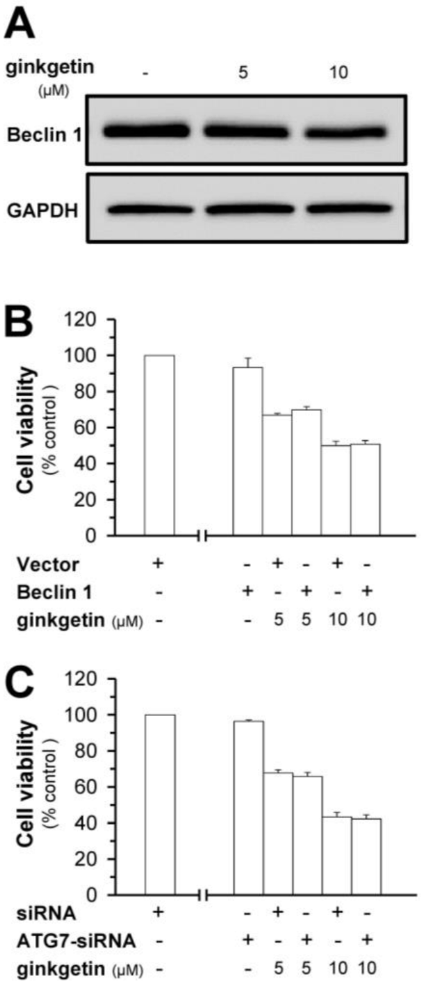

Supplementary Figure 8: Beclin 1 overexpression and ATG7 knockdown cannot rescue the cell viability in ginkgetin-treated cells. **(A)** A549 cells were treated with ginkgetin at 5 and 10 μM for 48 hours, the protein level of Beclin 1 (~60 kDa) was measured by western blot, GAPDH (~37 kDa) served as control. **(B)** A549 cells were seeded in 96-well plates (3×10^3 cells/well), allowed to adhere overnight and transfected with pReceiver-M98 or Beclin WT plasmid (Genecopoeia, Rockville, MD) (10 ng/well) for 4 hours, subsequently treated with ginkgetin for 48 hours. **(C)** A549 cells were seeded in 96-well plates (3×10^3 cells/well), allowed to adhere overnight and transfected with siRNA plasmid control, or ATG7 siRNA (CST, Danvers, MA), for 4 hours, subsequently treated with ginkgetin for 48 hours. Values are in percentage of cell survival rate. Each point represents mean ± SEM, *n*=3.

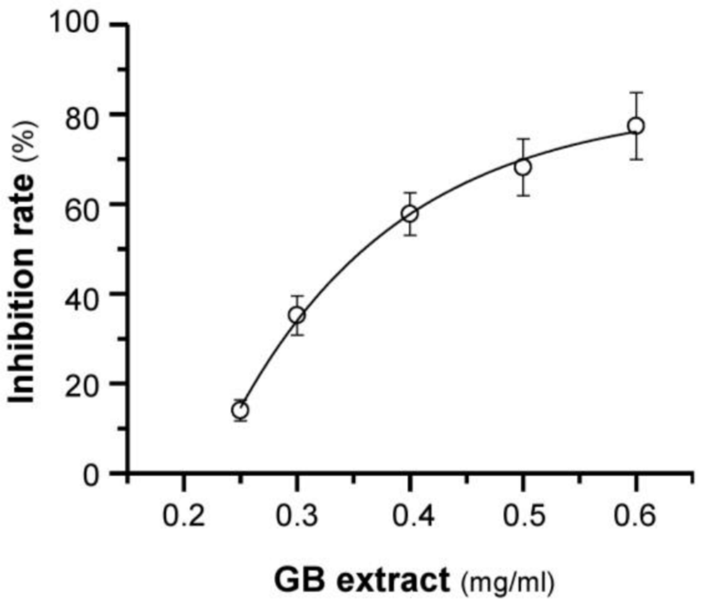

Supplementary Figure 9: *Ginkgo biloba* extract inhibits cell proliferation in A549 cell line. Cells were seeded in 96-well plates (3×10^3 cells/well) and allowed to adhere overnight and subsequently were treated with increasing concentration of *G. biloba* extract (prepared according to Chinese Pharmacopoeia) for 48 hours. Values are in percentage of cell growth inhibition. Results are expressed as the mean ± SEM, $n = 3$.
